# Cerebrospinal fluid soluble protein tyrosine phosphatase receptor type Z detected with human natural killer-1 antibody as a practical biomarker for glioma diagnosis

**DOI:** 10.1093/noajnl/vdag101

**Published:** 2026-04-16

**Authors:** Yu Naruse, Masazumi Fujii, Kenichiro Nagai, Ryo Hiruta, Toshie Sakagami, Takuru Kobayashi, Kazuto Takahashi, Junko Iijima, Hideaki Suzuki, Kasumi Hattori, Kazuaki Kanai, Yuka Oka, Yuko Hashimoto, Shunichi Koriyama, Taiichi Saito, Yoshihiro Muragaki, Takakazu Kawamata, Takashi Sasayama, Jun Yasuda, Miwa Uzuki, Yasushi Kawaguchi, Kentaro Kawata, Yoshiki Yamaguchi, Shiori Go, Hiromu Arakawa, Hiroyuki Kaji, Shinobu Kitazume

**Affiliations:** Department of Neurosurgery, Fukushima Medical University, Fukushima, Japan; Department of Neurosurgery, Fukushima Medical University, Fukushima, Japan; Department of Neurosurgery, Fukushima Medical University, Fukushima, Japan; Department of Neurosurgery, Fukushima Medical University, Fukushima, Japan; Department of Clinical Laboratory Sciences, Graduate School of Health Sciences, Fukushima Medical University, Fukushima, Japan; Department of Clinical Laboratory Sciences, Graduate School of Health Sciences, Fukushima Medical University, Fukushima, Japan; Department of Clinical Laboratory Sciences, Graduate School of Health Sciences, Fukushima Medical University, Fukushima, Japan; Department of Clinical Laboratory Sciences, Graduate School of Health Sciences, Fukushima Medical University, Fukushima, Japan; Department of Clinical Laboratory Sciences, Graduate School of Health Sciences, Fukushima Medical University, Fukushima, Japan; Department of Neurology, Fukushima Medical University, Fukushima, Japan; Department of Neurology, Fukushima Medical University, Fukushima, Japan; Department of Diagnostic Pathology, Fukushima Medical University, Fukushima, Japan; Department of Diagnostic Pathology, Fukushima Medical University, Fukushima, Japan; Department of Neurosurgery, Tokyo Women’s Medical University, Tokyo, Japan; Department of Neurosurgery, Tokyo Women’s Medical University, Tokyo, Japan; Department of Neurosurgery, Tokyo Women’s Medical University, Tokyo, Japan; Department of Neurosurgery, Tokyo Women’s Medical University, Tokyo, Japan; Department of Neurosurgery, Kobe University Graduate School of Medicine, Kobe, Japan; Division of Molecular and Cellular Oncology, Miyagi Cancer Center Research Institute, Natori, Japan; Department of Clinical Laboratory Sciences, Graduate School of Health Sciences, Fukushima Medical University, Fukushima, Japan; Department of Infectious Disease Control, The Institute of Medical Science, The University of Tokyo, Tokyo, Japan; Division of Molecular Virology, Department of Microbiology and Immunology, The Institute of Medical Science, The University of Tokyo, Tokyo, Japan; Research Center for Asian Infectious Diseases, The Institute of Medical Science, The University of Tokyo, Tokyo, Japan; Integrated Research Center for Self-Care Technology, National Institute of Advanced Industrial Science and Technology (AIST), Kagawa, Japan; Department of Medical Device Engineering, Kobe University Graduate School of Medicine, Kobe, Japan; Division of Structural Glycobiology, Tohoku Medical and Pharmaceutical University, Miyagi, Japan; Institute for Glyco-core Research (iGCORE), Nagoya University, Furo-cho, Chikusa, Nagoya, Japan; Institute for Glyco-core Research (iGCORE), Nagoya University, Furo-cho, Chikusa, Nagoya, Japan; Institute for Glyco-core Research (iGCORE), Nagoya University, Furo-cho, Chikusa, Nagoya, Japan; Department of Clinical Laboratory Sciences, Graduate School of Health Sciences, Fukushima Medical University, Fukushima, Japan

**Keywords:** biomarker, glioma, HNK-1, primary CNS lymphoma, PTPRZ

## Abstract

**Background:**

A major challenge in managing gliomas is the lack of reliable and clinically applicable biomarkers. Imaging alone is often insufficient to distinguish gliomas from primary central nervous system lymphoma (PCNSL), necessitating invasive biopsy. Soluble protein tyrosine phosphatase receptor type Z (sPTPRZ) in cerebrospinal fluid (CSF) has recently emerged as a promising candidate. We evaluated the diagnostic utility of CSF sPTPRZ in differentiating gliomas from PCNSL.

**Methods:**

We developed an anti-HNK-1 antibody-based ELISA for CSF sPTPRZ and analyzed 51 glioma, 15 PCNSL, and 20 control patients. Complementary assays included western blotting, immunohistochemistry, qPCR. sPTPRZ immunopurified from glioma CSF was used to determine HNK-1 epitope sites. RNA-seq data from the C-CAT database were used to identify PTPRZ gene fusions.

**Results:**

CSF sPTPRZ levels were significantly elevated in glioma patients compared with both controls and PCNSL, demonstrating robust discrimination between these entities (AUC = 0.910 vs controls; AUC = 0.805 vs PCNSL; *P* = .0010). PTPRZ expression was absent in PCNSL, consistent with low CSF sPTPRZ. Mass spectrometry localized the HNK-1 epitope to exon 12 of PTPRZ-long, absent in PTPRZ-MET fusion variants (2% of gliomas). These insights may explain low sPTPRZ levels in a subset of gliomas.

**Conclusion:**

CSF sPTPRZ detection with anti-HNK-1 antibody is a minimally invasive biomarker for glioma. It significantly improves diagnostic discrimination from PCNSL, and provides a molecular rationale for biomarker-negative glioma subsets with PTPRZ-MET fusions.

Key Points

- CSF sPTPRZ is a promising biomarker that distinguishes gliomas from PCNSL with good diagnostic performance.
- HNK-1 antibody enables accurate detection of sPTPRZ in glioma patients’ CSF.
- PTPRZ-MET fusion explains PTPRZ negativity in a subset of gliomas.

Importance of the Study

Gliomas pose numerous challenges in diagnosis and treatment, among which the lack of clinically applicable diagnostic biomarkers remains a major issue. sPTPRZ in CSF has emerged as a promising candidate biomarker. This study aimed to evaluate the diagnostic utility of CSF sPTPRZ in distinguishing gliomas from PCNSL, a differential diagnosis that is often difficult to achieve through imaging alone. We developed a sandwich ELISA using an anti-HNK-1 antibody and demonstrated that sPTPRZ levels were significantly higher in glioma patients than in those with PCNSL or in control subjects. In addition, we identified a PTPRZ-MET fusion as a potential cause of sPTPRZ negativity in a subset of gliomas. These findings suggest that detection of sPTPRZ via HNK-1 provides a minimally invasive, rapid, and accurate method to support glioma diagnosis, offering a favorable profile for differentiating gliomas from PCNSL. This approach may contribute to improved clinical decision-making and more personalized treatment strategies.

Gliomas are among the most common primary brain tumors and remain a challenging group of intractable cancers with significant obstacles in both diagnosis and treatment.^1–3^ Among the various challenges in clinical management, the absence of specific and clinically applicable fluid-based diagnostic biomarkers is, especially, a critical issue. Because imaging studies alone often fail to provide a definitive diagnosis, invasive procedures such as biopsy are sometimes required. To enable timely and accurate diagnosis that facilitates prompt therapeutic intervention, the development of noninvasive and rapid fluid-based diagnostic methods is urgently needed. Moreover, current imaging modalities are often insufficient for reliably assessing treatment response or detecting recurrence. Therefore, the identification of specific fluid biomarkers is highly desirable for improving disease monitoring and clinical decision-making.

PTPRZ is a membrane-bound protein that is highly expressed in central nervous system (CNS) glial cells, including oligodendrocyte precursor cells, astrocytes, and oligodendrocytes.^4^^,^^5^ It has been shown that PTPRZ is more highly expressed in glioma cells compared to normal brain tissue.^6–8^ Furthermore, the extracellular region is cleaved and shed, and the soluble form is known as sPTPRZ is markedly elevated in the cerebrospinal fluid (CSF) of glioma patients,^9^ suggesting that sPTPRZ may serve as a promising biomarker candidate for gliomas.

PTPRZ is heavily glycosylated with chondroitin sulfate,^10^ keratan sulfate,^11^ N-glycans, and unique human natural killer-1 (HNK-1) epitope consists of a sulfated trisaccharide^12^ is attached to the O-mannosyl core M2 glycans.^13^^,^^14^ So far, HNK-1-capped O-mannosyl glycans and core M2 glycans are only found in PTPRZ.^15^ In mice deficient in GnT-IX, a glycosyltransferase involved in the formation of the core M2 branching structure, a reduction in the HNK-1 epitope on PTPRZ has been observed.^14^ Furthermore, in a xenograft model using glioma cells with GnT-IX knockdown, a marked reduction in tumor growth was observed, indicating that the core M2 glycan carrying the HNK-1 epitope on PTPRZ plays a regulatory role in glioma tumorigenicity.^16^ PTPRZ has been implicated in glioma progression and may serve as a marker or therapeutic target for glioma.^9^^,^^16^^,^^17^ However, the detailed expression levels of sPTPRZ in the CSF remain unclear for other types of brain tumors, including primary central nervous system lymphoma (PCNSL), which is of particular clinical importance in the differential diagnosis of gliomas.

In this study, we evaluated whether CSF sPTPRZ could be useful in distinguishing glioma from PCNSL. To do this, we have developed sandwich ELISA with anti-HNK-1 antibody, which is shown to be specifically and highly reactive with CSF sPTPRZ. Our findings revealed that CSF sPTPRZ derived from tumors was more abundant in glioma patients than in controls and PCNSL patients. This study suggests that CSF sPTPRZ has potential as a liquid biopsy marker for minimally invasive differentiation between glioma and PCNSL.

## Methods

### Ethics

This study was approved by the ethics committee of Fukushima Medical University (approval numbers 2020-287 and 29378), which is guided by local policy, national laws, and the World Medical Association Declaration of Helsinki.

### Human Samples

CSF samples and tumor specimens were collected at Fukushima Medical University, Tokyo Women’s Medical University, and Kobe University. CSF samples were collected during surgery without deviating from the normal procedures. After removal of cells and debris, CSF samples were stored at −80 °C until analysis. Tumor specimens collected upon biopsy or tumor removal, fixed with formalin, embedded in paraffin, and sliced into 4-µm-thick sections for Hematoxylin and Eosin or immunohistochemical staining. Tumors were diagnosed and graded according to the current WHO Classification.^3^ The control subjects were nontumor disorders, including idiopathic normal pressure hydrocephalus, unruptured cerebral aneurysms, facial spasm, or trigeminal neuralgia. The clinical profiles of the patients are summarized in Table 1.

**Table 1.** Clinical characteristics and diagnostic classification of study cohort by tumor or disease type

Tumor/disease type	No. of patients
**Glioma**	**51**
Glioblastoma, IDH-wildtype, WHO grade 4	16
Oligodendroglioma, IDH-mutant and 1p/19q-codeleted, WHO grade 3	2
Oligodendroglioma, IDH-mutant and 1p/19q-codeleted, WHO grade 2	10
Astrocytoma, IDH-mutant, WHO grade 4	2
Astrocytoma, IDH-mutant, WHO grade 3	7
Astrocytoma, IDH-mutant, WHO grade 2	2
Diffuse pediatric-type high-grade glioma, H3-wildtype and IDH-wildtype, CNS WHO grade 4	2
Diffuse midline glioma, H3 K27-altered, CNS WHO grade 4	1
Others	9
**PCNSL**	**15**
Primary diffuse large B-cell lymphoma	15
**Control**	**20**
Unruptured cerebral aneurysm	7
Trigeminal neuralgia	5
Hemifacial spasm	3
Atherosclerotic intracranial arterial stenosis	3
Idiopathic normal pressure hydrocephalus	1
Spastic quadriplegic cerebral palsy	1

### Materials

Anti-SGPG (HNK-1) mouse monoclonal IgG antibody (A2706) is a generous gift from TCI Chemicals. The following antibodies were purchased: anti-PTPRζ (catalog no.: sc-33664; Santa Cruz Biotechnology, referred to as “anti-PTPRZ [Santa Cruz]”), Cat-315 (catalog no.: MAB1581; Merck Millipore) mouse IgM, anti-transthyretin (ab9015; Abcam, referred to as “anti-TTR”) polyclonal sheep IgG, anti-3-beta-glucuronosyltransferase 1, (GlcAT-P; catalog no.: ab199156; abcam), anti-CD3 (ready-to-use product (RTU), clone LN10, Leica Biosystems, UK), anti-CD-10 (RTU, clone 56C6, Leica Biosystems, United Kingdom), anti-CD20 (RTU, clone L26, Leica Biosystems, UK), anti-Ki-67 (RTU, clone MM1, Leica Biosystems, United Kingdom) and antihistone H3 (catalog no.: H0164; Sigma-Aldrich) rabbit IgG. The following horseradish peroxidase-conjugated secondary antibodies were used: goat antimouse IgM (catalog no.: SAB-110; StressGen), and goat anti-mouse IgG (catalog no.: NA931-1ML; Cytiva). The following fluorescently labeled secondary antibodies were used: Alexa Fluor 546 goat antimouse IgM (catalog no.: A21045; Life Technologies).

### Cell Culture

HEK293FT cells (Thermo Fisher), human glioblastoma LN-229 (CRL-2611; ATCC), U-251 (9063001, Sigma-Aldrich), U-118 (HTB-15; ATCC), anaplastic glioma NP-2 (RCB4498, RIKEN Cell Bank) were maintained in DMEM (D5796; Sigma) containing 10% FBS (Equitech-Bio, Inc). Human primary CNS lymphoma TK (JCRB1206, JCRB Cell Bank), HKBML (RCB0820, RIKEN Cell Bank) and Burkitt’s lymphoma Raji (CCL-86, ATCC) were maintained in RPMI-1640 (R8758; Sigma) containing 10% FBS. Both media were supplemented with penicillin-streptomycin solution (catalog no.: 168-23191; FUJIFILM). GlcAT-P was expressed in a series of glioma and lymphoma cell lines as previously reported.^16^

### Western Blot Analysis (CSF)

CSF samples (20 µL) were digested with 0.4 mU Chondroitinase ABC (Sigma) in Tris-acetate buffer (pH 7.4) containing protease inhibitor cocktail (Nacalai) for 1 h at 37 °C. The samples were subjected to SDS-PAGE (3%-10% gradient gels; Atto) and were transferred to nitrocellulose membranes. After blocking with 5% nonfat dried milk in TBS buffer (pH 7.4) containing 0.1% Tween-20 for 1 h, the membranes were probed with anti-PTPRZ (Santa Cruz; 1:1000 dilution), anti-HNK-1 (1:1000 dilution), or anti-TTR (1:1000 dilution) antibodies overnight at 4 °C, and with the appropriate horseradish peroxidase-conjugated secondary antibodies (1:3000 dilution) for 1 h at 25 °C. The blots were developed using SuperSignal West Femto maximum sensitivity substrate (code: 34096; Thermo Fisher Scientific) for PTPRZ (Santa Cruz) and MET, and ECL Western Blotting Detection Reagent (code: RPN2106; GE Healthcare) for HNK-1 and TTR. Signals were detected with the ChemiDoc Touch MP (Bio-Rad) and quantified using Image Lab Software (Bio-Rad).

### Immunohistochemistry and Histology

Paraffin sections of gliomas and PCNSL sliced into 4 µm thickness were obtained from Fukushima Medical University Hospital. The sections were baked at 60 °C for 30 min, deparaffinized in xylene, rehydrated in an ethanol series (100%, 90%, 80%, and 70%), and heated with Histofine antigen retrieval regent, pH9 (code: 415211; Nichirei biosciences) for 15 min, and leaved at room temperature for more than 20 min to cool slowly. Following blocked with 5% goat serum in PBS for 15 min, sections were incubated with primary antibodies (Cat-315, 1:250 dilution; anti-HNK-1, 1:100 dilution) for 1 h at 25 °C and with fluorescently labeled secondary antibodies (1:300 dilution) for 45 min at 25 °C. After washing, VECTASHIELD Vibrance Antifade Mounting Medium (code: H-1800; Vector Laboratories) was applied to sections. Staining for CD20, CD3, CD10, and Ki-67 was performed using standard retrieval on an automated staining machine (Leica BOND-III system, Leica Biosystems, Australia). The images were captured using a fluorescence microscope BZ-X810 (Keyence).

### Immunoprecipitation

Digestion of CSF with chondroitinase ABC (Sigma) was performed in phosphate buffer (pH 7.4), as necessary. The CSF samples (20 µL) were incubated with anti-HNK-1 antibody for 2 h at 4 °C on a rotator, and incubated with Dynabeads protein G (DB1003D, Thermo Fisher Scientific) for 30 min at 4 °C on a rotator. The beads were washed with PBS and used for western blotting. For the purification of CSF sPTPRZ for LC-MS/MS analysis, anti-HNK-1 antibody coupled to Dynabeads protein G with DMP (Thermo Fisher Scientific) was incubated with glioma CSF (2 mL). The sPTPRZ-bound beads were washed with PBS and boiled with 40 μL phase transfer surfactant buffer (PTS-buffer, 12 mM sodium deoxycholate and 12 mM sodium N-lauroylsarcosinate in 50 mM Hepes [pH 8.0])^18^ and the eluate was used for LC-MS/MS analysis.

### sPTPRZ ELISA

An anti-HNK-1 antibody was conjugated with alkaline phosphatase using AP conjugation kit (abcam, ab102850). A 96-well immunoplate (96 well MicroWell MaxiSorp, Nunc) was coated with anti-HNK-1 antibody (10 µg/mL in 0.1M sodium-carbonate buffer, pH9.5) and immobilized overnight at 4 °C. After washing the plates with PBS 2 times, the plate was incubated with 1% BSA in PBS and immobilized overnight at 4 °C. After washing the plates with PBS 5 times, recombinant sPTPRZ754 bearing the HNK-1 epitope was used as a standard,^16^ and CSF samples diluted 4-fold with EIA buffer (27736D, IBL) were added. The plate was then incubated at 37 °C for 30 min. After washing the plates with PBS 8 times, AP-conjugated anti-HNK-1 antibody (1 µg/mL) was incubated for 30 min at 37 °C. As an AP colorimetric substrate, 1-Step PNPP (37621; Thermo Fisher Scientific) was used. The optical density was measured at 405 nm (OD_405_) using Nivo 3F plate reader (Perkin Elmer Victor).

### Real Time PCR Analysis

Glioma tissues were homogenized with 4 mL of TriPure Isolation Reagent (Roche, 11667165001) with gentleMACS Dissosiator (RNA_02 program, Miltenyl Biotec). The RNA pellets were suspended with PBS and further purified with a High Pure RNA Isolation Kit (Roche, 11828665001). For the cells cultured *in vitro*, only a High Pure RNA Isolation Kit was used for RNA isolation. RNA samples (up to 5 μg) were then reverse-transcribed with random hexamers using a Transcriptor First-Strand cDNA Synthesis Kit (Roche, 04379012001) as per the manufacturer’s protocol. The amount of cDNA of specific genes was then quantified using a TaKaRa qPCR probe (TaKaRa) or the Universal ProbeLibrary (Roche) with TaqMan Master (Roche) and a LightCycler 96 system (Roche) in accordance with the manufacturers’ instructions. The primer and probe sequences are shown in Supplementary Table S1. The levels of mRNA were normalized to the corresponding ribosomal RNA levels and calculated using the comparative cycle threshold (2−ΔΔCt) method.^19^

### LC-MS/MS Analysis

Enriched sPTPRZ in PTS-buffer was digested with endoproteinase Lys-C (Promega, United States) followed by Trypsin (Promega) after reduction with dithiothreitol (Thermo Fisher Scientific, USA) and carbamidomethylation with iodoacetamide (Thermo). The digests were desalted using GL-Tip SDB (GL Science, Japan) and freeze-dried. The peptides were treated with Peptide-N-glycosidase F (1 mU, Takara, Japan) and O-glycopeptides were captured by hydrophilic interaction chromatography (HILIC) on an Amide-80 column (TOSOH, Japan). Obtained O-glycopeptides were analyzed by LC/MS/MS using UltiMate 3000 RSLCnano-Orbitrap Eclipse tribrid mass spectrometer system (Thermo Fisher Scientific). Peptides were separated using C18 column (inner diameter: 0.075 mm, length: 25 cm, 1.9 µm particle, Nikkyo Technos, Japan) by the gradient of acetonitrile (3%-35%, 45 min, 300 nL/min) in 0.1% formic acid. Eluate was ionized by electrospray method at 2 kV and introduced into the mass spectrometer. Mass spectra were obtained by data-dependent acquisition and positive ion mode. Both precursor-survey MS and fragment mass (MS/MS) spectra were measured by orbitrap analyzer at the resolution of 120 K and 60 K, respectively. Selected precursor ions were fragmented by higher-energy collision-induced dissociation method (stepped collision energy of 20%, 30%, and 40%). Obtained MS/MS spectra were searched using Byonic (ProteinMetrics, United States) using glycan DB containing 100 glycan compositions including in part HexA or HNK-1 (HexNAc-Hex-HexA-Sulfo). Mass tolerance of MS and MS/MS were 2 ppm and 5 ppm, respectively. Peptide N-terminal pyro-Glutamine (Gln), Peptide N-terminal Ammonia loss (Cys), deamidation (Asn), and oxidation (M) were considered as variable modifications. Protein database used was SwissProt_Human fasta file (downloaded on May 19, 2020, 40,000 entries). The results were processed with Proteome Discoverer (ver.2.5, Thermo Fisher Scientific). The data showing “Confidence”=“High” and glycosylation were treated as “an identified glycopeptide.”

### C-CAT Data Analysis

We accessed C-CAT database on January 29 and 30, 2025 (in total 88,688 cases were registered on January 30) and 1938 cases of gliomas were registered in the database. To efficiently collect the PTPRZ1 fusion cases, we selected the cases that were analyzed with GenMineTOP, a comprehensive genomic panel whose RNA-seq data were confirmed superiority on the DNA panel in the detection of fusion genes.^20^ We obtained 301 glioma cases and 6 cases showed the PTPRZ1-MET fusion gene products.

### Bioinformatic Analysis

Comprehensive gene expression profiles of lymphoma (GEO accession GSE155398^21^) and glioma (GEO accession GSE266210^22^) were obtained from RNA-seq data published in the GEO database.^23^^,^^24^ For the quantification of gene expression profiles, we used the NCBI-generated data on which the estimated gene expression abundances were precalculated using the pipeline built by the NCBI SRA and GEO teams. Briefly, the RNA-seq data are aligned to the reference genome (GRCh38; GCA_000001405.15) using HISAT2^20^ and the raw counts of those where more than 50% of the reads are aligned are quantified using Subread featureCounts^25^ with *Homo sapiens* annotation data (release 109). The gene expression profile in each sample was quantified as transcripts per million (TPM) from the raw count data. Among the genes whose expression was precomputed, the estimated expressions abundances for PTPRZ and GnT-IX were extracted.

### Statistical Analysis

Data are presented as the mean ± SEM. Given the relatively small sample size and the inability to assume a normal distribution, nonparametric tests were used. Specifically, the Mann-Whitney U test was applied for comparisons between 2 groups, and the Kruskal-Wallis test followed by Dunn’s post hoc test was used for multiple-group comparisons. Data were analyzed using Microsoft Excel and GraphPad Prism 10.6.1 (Statcon).

### Data Availability

The LC-MS/MS data generated in this study have been deposited in jPOST database under accession number [JPST003723/PXD062244]. Other datasets generated or analyzed during the current study are available from the corresponding author S. Kitazume upon reasonable request. Supplementary Material is available at *NOA* online.

The LC-MS/MS data have been deposited in the jPOST database under accession number JPST003723/PXD062244. Other data supporting the findings of this study are available from the corresponding author upon reasonable request. This study was approved by the ethics committee of Fukushima Medical University (approval numbers 2020-287 and 29378) and conducted in accordance with the Declaration of Helsinki.

## Results

### Patient Cohort

**Figure 1.**
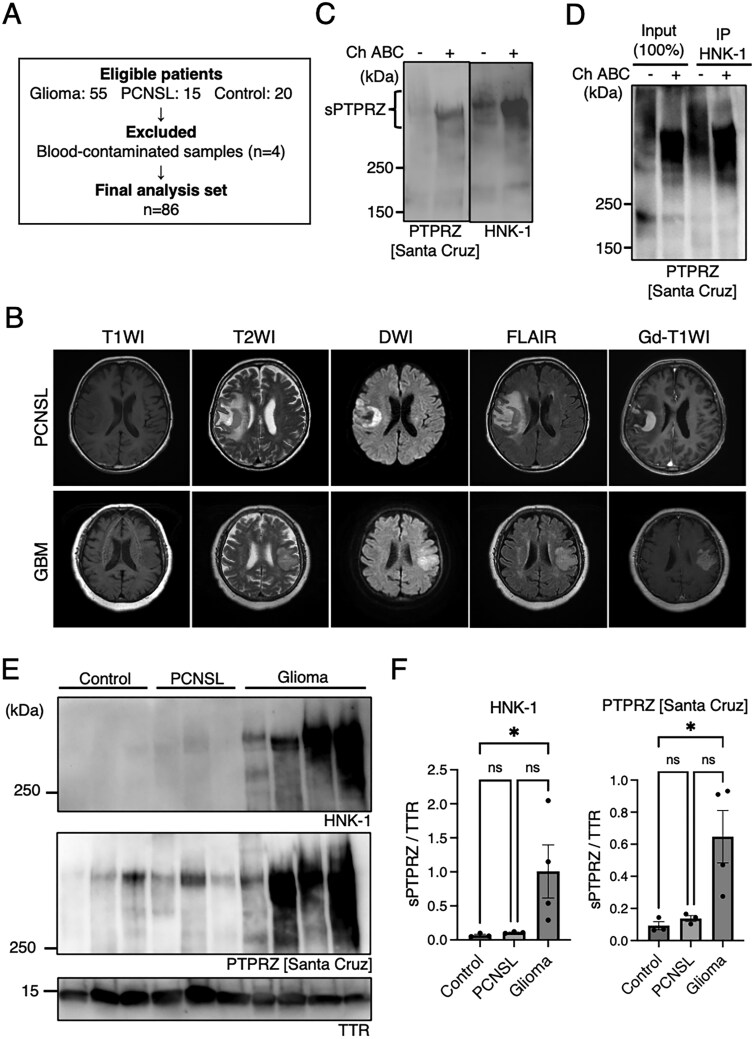
Detection of sPTPRZ in CSF using the anti-HNK-1 antibody. (A) Flowchart showing the selection of eligible patients and the final analysis set for CSF samples. (B) Representative MRI images of a PCNSL and a glioblastoma, IDH-wildtype, WHO grade 4, with overlapping imaging ­characteristics. (C) Western blot analysis comparing the detection of sPTPRZ in CSF using anti-HNK-1 and anti-PTPRZ [Santa Cruz] antibodies. (D) Immunoprecipitation of sPTPRZ from CSF using anti-HNK-1 antibody, followed by detection with anti-PTPRZ [Santa Cruz] antibody. (E) Western blot analysis of sPTPRZ levels in CSF samples from glioma, PCNSL, and control patients. (F) Quantification of sPTPRZ levels in glioma, PCNSL, and control samples. Data are presented as mean ± SEM. Statistical significance was determined using the Kruskal-Wallis test followed by Dunn’s multiple-comparisons test (GraphPad Prism 10.6.1).

A total of 90 patients (glioma, *n* = 55; PCNSL, *n* = 15; controls, *n* = 20) were initially enrolled. After excluding blood-contaminated samples (*n* = 4), 86 samples were included in the final analysis. The patient selection process is summarized in Figure 1A.

### Analysis of CSF sPTPRZ With Anti-HNK-1 Antibody

MRI findings for gliomas and PCNSLs can sometimes overlap, making differential diagnosis challenging. Figure 1B shows MRI images of a typical PCNSL and a glioblastoma with MRI findings resembling those of PCNSL. The MRI images of glioblastoma demonstrated hypointense on T1-weighted imaging, hyperintense on T2-weighted imaging, hyperintense on diffusion-weighted imaging, hyperintense on fluid attenuated inversion recovery (FLAIR) imaging, and homogenous enhancement on gadolinium-enhanced imaging, requiring a tissue biopsy for differentiation. To clarify if CSF sPTPRZ could be useful in distinguishing glioma from PCNSL, we first performed western blotting using CSF from glioma and PCNSL patients, and control samples. Most of commercially available anti-PTPRZ antibodies do not detect heavily glycosylated CSF sPTPRZ and limited anti-PTPRZ antibodies, such as those from Santa Cruz, require chondroitinase ABC (ChABC) treatment to enable detection, and these anti-PTPRZ IgM antibodies recognize the HNK-1 epitope on the O-mannosyl glycans of sPTPRZ.^16^^,^^26^ Therefore, we here first examined the utility of the anti-HNK-1 IgG antibody (anti-SGPG, TCI Chemicals)^27^ for detecting CSF PTPRZ. When CSF from the same glioma patient was tested, the anti-HNK-1 antibody displayed a stronger signal as compared with the anti-PTPRZ (Santa Cruz) antibody (Figure 1C). The anti-HNK-1 antibody showed a signal even in CSF without ChABC treatment. We also confirm that the anti-HNK-1 antibody dose recognize sPTPRZ, since the CSF immunoprecipitants with the anti-HNK-1 antibody were clearly detected with the anti-PTPRZ (Santa Cruz) antibody, in which the signal intensity of the immunoprecipitants was equivalent to 100% of the input (Figure 1D). In the brain, α-amino-3-hydroxy-5-methyl-4-isoxazolepropionic acid (AMPA)-type glutamate receptor (AMPAR) subunit GluA2 is another membrane-bound glycoproteins bearing the HNK-1 epitope on the N-glycans.^28^^,^^29^ However, since no distinct signal was observed around 100 kDa, corresponding to GluA2, it is considered that shedding of HNK-1-bound GluA2 into the CSF is negligible. These findings indicate that the anti-HNK-1 antibody has high affinity to CSF sPTPRZ, and we next used anti-HNK-1 and anti-PTPRZ (Santa Cruz) antibodies to evaluate sPTPRZ levels in CSF samples from glioma, PCNSL, and control groups. Strong sPTPRZ signals were observed in 3 out of 4 glioma samples with both antibodies, whereas in the control and PCNSL samples sPTPRZ signals were weak with anti-PTPRZ (Santa Cruz) and these signals were not even detectable with anti-HNK-1 antibody (Figure 1E). One glioma sample showed markedly reduced sPTPRZ signal, indicating heterogeneity among glioma cases. Semiquantitative western blot analysis demonstrated that sPTPRZ levels were significantly higher in CSF from glioma patients compared with control CSF (Figure 1F). Although sPTPRZ levels were also higher in glioma patients than in PCNSL patients, this difference was not statistically significant, likely reflecting the limited sample size of this exploratory cohort.

### PTPRZ Protein Expression in Glioma and PCNSL

**Figure 2.**
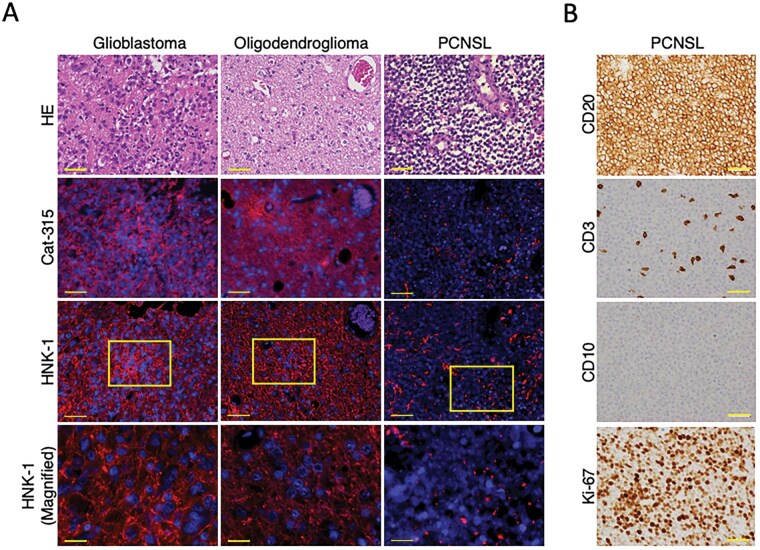
HNK-1 expression in glioma tissues. (A) Immunofluorescent images of brain sections from patients with glioblastoma, IDH-wildtype, WHO grade 4, oligodendroglioma IDH-mutant and 1p/19q-codeleted, WHO grade 2, and primary diffuse large B-cell lymphoma (not included in CSF analysis). Sections were stained with the Cat-315 (red) or HNK-1 (red) antibodies, and for DAPI (blue). Scale bar: 50 µm or 20 µm (magnified area). (B) Immunohistochemical images of primary diffuse large B-cell lymphoma stained for CD20, CD3, CD10, and Ki-67. Scale bar: 50 µm.

Low sPTPRZ levels in CSF from PCNSL patients suggests reduced PTPRZ expression in PCNSL tumor cells. To clarify this, we conducted histological examination and immunohistochemical staining of pathological sections from glioma (oligodendroglioma, IDH-mutant and 1p/19q-codeletion, WHO Grade 2; and glioblastoma, IDH-wild type) and PCNSL (diffuse large B cell lymphoma). We here used anti-HNK-1 and Cat-315 antibodies, the latter of which recognizes the HNK-1 epitope plus the PTPRZ peptide region.^26^^,^^30^ H&E staining revealed characteristic features of each tumor (Figure 2A): glioblastomas exhibited frequent mitoses and necrosis, densely proliferating atypical cells^31^; oligodendrogliomas displayed “fried-egg”-like tumor cells^32^; PCNSLs showed diffuse infiltration of large tumor cells with round, indented, or cleaved nuclei, prominent nuclei, and CD20/CD10 positivity, CD3 negativity, and a Ki-67 index of 80%, consistent with diffuse large B-cell lymphoma^33^ (Figure 2B). Glioblastoma and oligodendroglioma tumor cells were positive for both Cat-315 and anti-HNK-1 antibodies in the cytoplasm, whereas PCNSL tumor cells showed no reaction with either antibody. Several Cat-315- and HNK-1-positive signals observed in PCNSL samples appeared to originate from surrounding glial tissue, as suggested by the co-localization of HNK-1 with GFAP in double immunostaining (Supplementary Figure S1). These findings support the interpretation that the HNK-1 epitope detected in this study reflects PTPRZ expressed in glial cells rather than in PCNSL tumor cells.

### Analysis of PTPRZ RNA Levels in Glioma and PCNSL Cells

**Figure 3.**
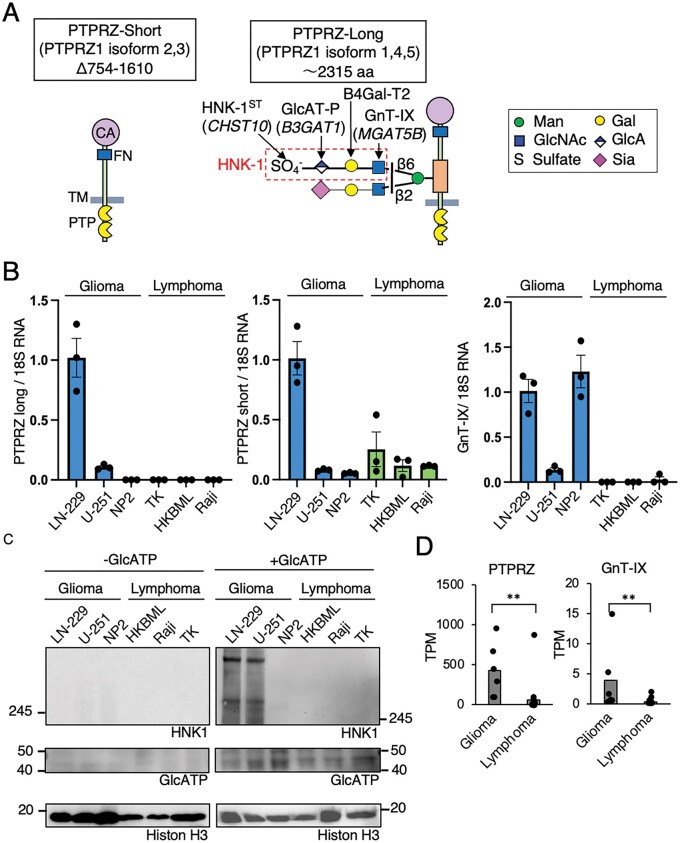
PTPRZ mRNA and protein expression in glioma and PCNSL. (A) Schematic representation of PTPRZ isoforms and their alternative splicing patterns. (B) Quantitative PCR analysis of PTPRZ mRNA expression in glioma (LN-229, NP2, U-251) and lymphoma-derived (TK, HKBML, and Raji) cell lines. (C) Western blot analysis of PTPRZ protein expression in glioma and lymphoma cell lines with and without GlcAT-P transduction. (D) RNA-seq analysis comparing PTPRZ and GnT-IX mRNA expression in glioma and PCNSL tumor tissues from the GEO database. Data are presented as mean ± SEM. Statistical analysis was performed using the Mann-Whitney U test (Microsoft Excel).

The detection of sPTPRZ in CSF and immunohistochemical analysis we have conducted both rely on antibodies that recognize the HNK-1 glycan chain of PTPRZ. The absence of PTPRZ in PCNSL cannot rule out the possibility that PTPRZ is expressed but lacks HNK-1 glycan chains. We then examined the mRNA expression levels of PTPRZ and the brain-specific glycosyltransferases GnT-IX, involved in the HNK-1 epitope formation, in glioma and PCNSL. Since PCNSL tumor samples are typically collected in minimal amounts solely for diagnostic purposes, sufficient tissue for conducting transcriptome analysis is not available. Therefore, we first performed qPCR analysis using cultured cell lines derived from glioma (LN-229, NP2, and U-251), PCNSL (TK, HKBML), and lymphoma (Raji). PTPRZ has multiple mRNA isoforms due to alternative mRNA splicing, and in humans, there are 2 main groups: PTPRZ-long and PTPRZ-short (Figure 3A).^34^ High level of CSF sPTPRZ in glioma patients is derived from PTPRZ-long.^9^ We found that both LN-229 and U-251 cells expressed significant levels of PTPRZ long form mRNA, whereas it was barely detectable in NP2 cells (Figure 3B). In the PCNSL and lymphoma-derived cell lines, expression of PTPRZ-long form was undetectable. While, PTPRZ-short mRNA is expressed at higher level in LN-229 and at a lower level in other cell types. GnT-IX eoncoding MGAT5B was expressed in all glioma-derived cell lines examined, but was not detected in PCNSL or lymphoma-derived cell lines. Next, we analyzed the PTPRZ protein levels in these cultured cell lines. It has previously been shown that mRNA expression of GlcAT-P, which is essential for the synthesis of the HNK-1 epitope, is significantly reduced *in vitro* cell culture conditions.^16^ Therefore, each cultured cell line was transduced with a retroviral vector to achieve ectopic expression of GlcAT-P, enabling the detection of PTPRZ using the HNK-1 antibody. Indeed, without GlcAT-P expression, PTPRZ was undetectable in the lysates from all cell lines, including glioma (Figure 3C). When GlcAT-P was expressed, PTPRZ protein was detected in glioma cell lines LN229 and U-251; however, it was not detectable in glioma NP2 cells or in any of the lymphoma cell lines, consistent with the qPCR results. Furthermore, RNA-seq data of tumor tissues derived from glioma (GSE266210) and PCNSL (GSE155398) were obtained from GEO, and we found that both *PTPRZ1* and *MGAT5B* mRNA levels were significantly higher in glioma samples compared to PCNSL samples (Figure 3D). Taken together, these findings indicate that glioma exhibits markedly higher PTPRZ mRNA expression compared to PCNSL. Additionally, GnT-IX, previously reported as a therapeutic target for glioma,^16^ was also found to be highly expressed in glioma.

### Quantitative Analysis of sPTPRZ in the CSF Among Glioma, PCNSL and Control Patient

**Figure 4.**
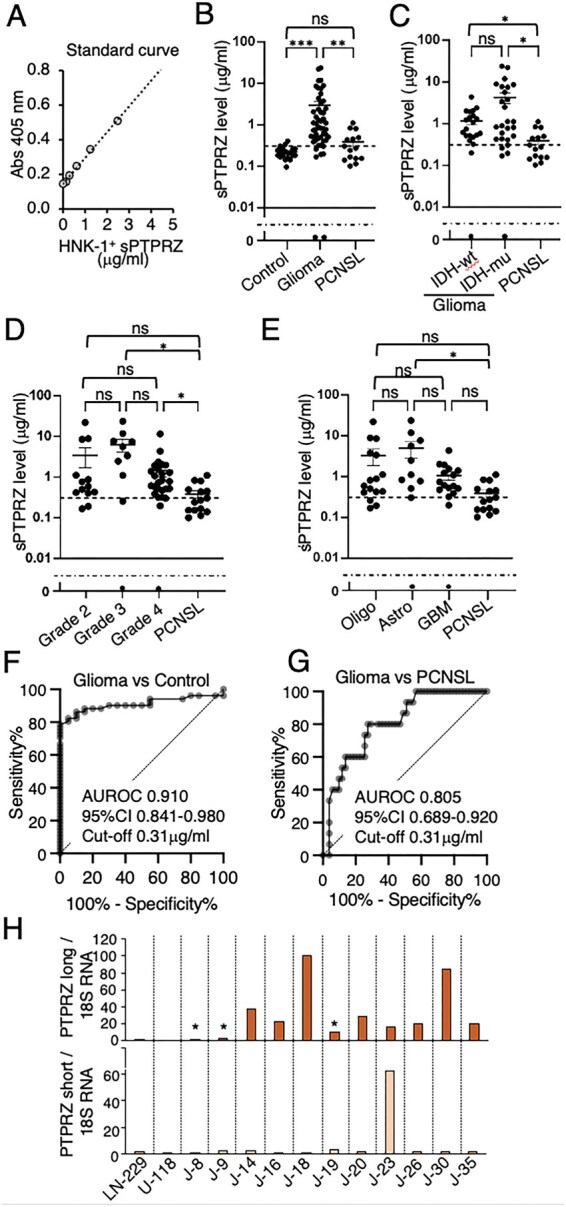
Quantitative analysis of CSF sPTPRZ in glioma, PCNSL, and control patients using a sandwich ELISA. (A) Standard curve for the newly developed sPTPRZ ELISA, demonstrating linearity of the assay. (B) CSF sPTPRZ levels in control (*n* = 20), glioma (*n* = 51), and PCNSL (*n* = 15) patients. (C) sPTPRZ levels according to IDH status in glioma (IDH-wild type [IDH-wt] and IDH-mutant [IDH-mut] and compared with PCNSL. (D) sPTPRZ levels according to glioma grade (grade 2, grade 3, grade 4 gliomas) and compared with PCNSL. (E) Comparison of CSF sPTPRZ levels among oligodendroglioma (Oligo), astrocytoma (Astro), glioblastoma (GBM), and PCNSL. For panels (B-E), statistical comparisons among groups were performed using the Kruskal-Wallis test followed by Dunn’s multiple-comparisons test (GraphPad Prism 10.6.1). Adjusted *P* values are reported. The horizontal dotted line does not represent a biological cutoff or detection limit but was included solely to facilitate visualization on the logarithmic scale. The dashed line represents the cutoff value (0.31 µg/ml). *P < .05, **P < .01, ***P < .001. (F) Receiver operating characteristic (ROC) curve analysis comparing glioma and control groups. (G) ROC curve analysis comparing glioma and PCNSL groups. The diagonal dashed line indicates chance-level performance. (I) Quantitative PCR analysis of long and short isoforms of PTPRZ mRNA normalized to 18S rRNA in glioma tissues. Asterisks in panel (H) indicate samples with CSF sPTPRZ levels below the cutoff threshold (0.31 μg/mL) as determined by ELISA.

Several lines of data shown in Figure 1 indicate that the anti-HNK-1 antibody possesses high specificity and sensitivity for detecting CSF sPTPRZ. We then used the anti-HNK-1 antibody as both the capture and detection antibodies and established a sandwich ELISA system. For the standard, recombinant sPTPRZ754 protein modified with HNK-1 glycan was used,^16^ yielding linearity and confirming the assay’s performance (Figure 4A). Using this sandwich ELISA, we quantified CSF sPTPRZ levels in 86 samples, including 51 glioma cases, 15 PCNSL cases, and 20 control cases (Table 1). CSF sPTPRZ levels were significantly elevated in glioma patients compared with both PCNSL (*P* = .0010) and control groups (*P* < .000001) (Figure 4B). Subgroup analysis within the control cohort revealed no single diagnosis with disproportionately elevated CSF sPTPRZ levels (Supplementary Figure S2). CSF sPTPRZ levels were elevated across glioma cases irrespective of IDH mutation status (Figure 4C) and tumor grade (Figure 4D). However, in grade-specific comparisons, a statistically significant difference was not observed between grade 2 glioma and PCNSL. In subtype-specific analyses, a statistically significant difference was observed between astrocytoma and PCNSL, whereas comparisons between PCNSL and other glioma subtypes did not reach statistical significance (Figure 4E). The receiver operating characteristic (ROC) curve for differentiating glioma from controls showed an area under the curve (AUC) of 0.910 (95% CI: 0.841-0.980). At a cutoff value of 0.31 µg/mL, sensitivity and specificity were 82% and 95%, respectively (Figure 4F). ROC curve analysis yielded an AUC of 0.805 (95% CI: 0.689-0.920) for discrimination between glioma and PCNSL (Figure 4G). Classification performance at the prespecified cutoff is summarized in Supplementary Table S2. Additionally, precision-recall analysis yielded an AUPRC of 0.937 (positive class: glioma), exceeding the baseline precision of 0.77 (Supplementary Figure S3). At the same threshold (0.31 μg/mL), the sensitivity and specificity were 0.86 and 0.60, respectively. The positive predictive value (precision) was 0.88 (Table S2). It should be noted that 14% of the CSF samples from glioma patients exhibited sPTPRZ levels below the cutoff value, consistent with findings in glioma cell lines such as NP2, which showed minimal PTPRZ expression.

We next performed qPCR analysis on glioma samples with high CSF sPTPRZ levels (*n *= 8) and those with levels below the cutoff (*n* = 3), including LN-229, a cell line known for relatively high PTPRZ expression (Figure 3B), and U-118, in which a PTPRZ-MET gene fusion has been reported.^35^ In glioma samples with high CSF sPTPRZ levels, the average PTPRZ mRNA expression was 40 times higher compared to LN-229 (Figure 4H). In contrast, glioma samples with CSF sPTPRZ levels below the cutoff exhibited significantly lower PTPRZ expression.

### PTPRZ-MET Gene Fusion Protein Lacks HNK-1 Epitope Region

**Figure 5.**
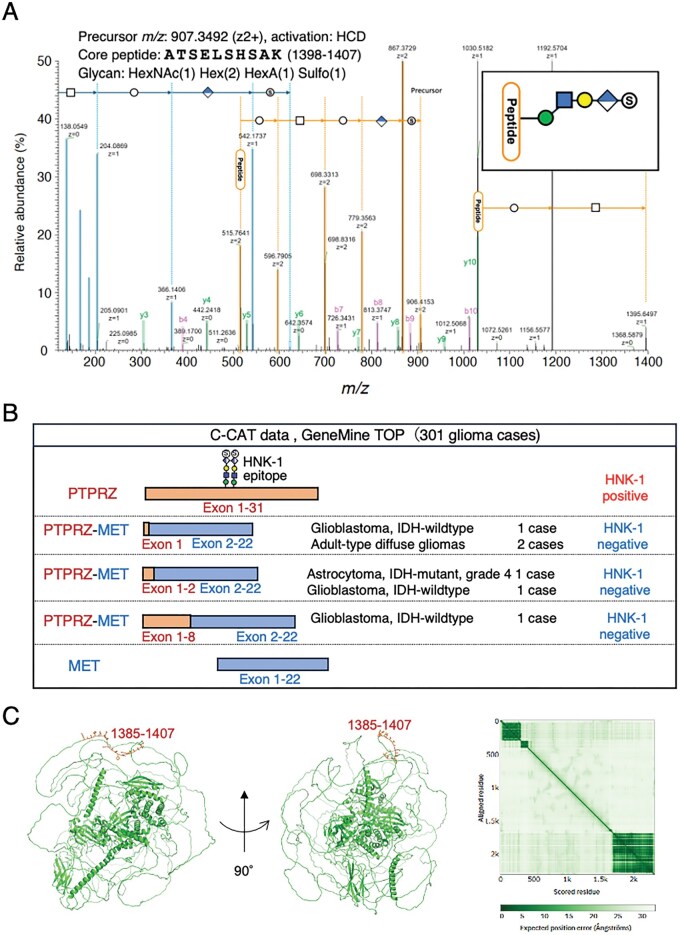
PTPRZ-MET fusion and HNK-1 glycosylation site mapping. (A) Annotated tandem mass (MS/MS) spectrum of an HNK-1-carrying glycopeptide, in which core peptide is ATSELSHSAK (1398-1407). After Peptide-N-glycosidase treatment, O-glycopeptides were obtained by HILIC from the digest after and analyzed by LC/MS. (B) Schematic representation of PTPRZ-MET gene fusion variants identified in glioma samples from the C-CAT database. The LC-MS/MS analysis of sPTPRZ shown in (B) revealed that the HNK-1 epitope specifically binds to the juxtamembrane region (exon 12) of the long form of PTPRZ. (C) AlphaFold-based structural model of PTPRZ highlighting the predicted HNK-1 glycosylation sites (1385-1407) in the disordered region.

Recently, accumulating reports have described glioma cases with several types of PTPRZ gene fusion, in which most of PTPRZ protein part is replaced with oncogenic proteins, such a MET, ETV1, and BRAF.^35–38^ Although the presence of the HNK-1 epitope depends on its site of addition, gene fusion products that lack most of the PTPRZ mRNA are likely to be PTPRZ-negative. To determine the localization of the HNK-1 epitope on nonfusion sPTPRZ, we performed site mapping using mass spectrometry. sPTPRZ was immunopurified from glioma patient CSF using an anti-HNK-1 antibody, digested with endoproteinase Lys-C followed by trypsin, and the enriched glycopeptides were treated with peptide-N-glycosidase F. O-glycopeptides were captured again and used for LC/MS analysis. Glycopeptides were identified from their higher energy collision-induced dissociation spectra by database search using Byonic with a glycan DB including GlcA-containing glycan compositions. Two glycopeptides, DGSVTSTKLLFPSK (1384-1397, Supplementary Figure S4) and ATSELSHSAK (1398-1407, Figure 5A) were found to have GlcA-containing glycan, HexNAc(2)Hex(2)GlcA(1)Sulfo(1) and HexNAc(1)Hex(2)GlcA(1)Sulfo(1), respectively. Their spectra indicate that hexose is attached first to each peptide and further extension of HexNAc-Hex-HexA-sulfate occurred at both sites. Presumed glycan structures are shown in the inset of the figures. The result indicates that O-Man glycans capped with HNK-1 epitope attach to exon 12, correspond to the juxtamembrane region unique to the long isoform. This finding aligns with our previous observation that the short isoform of sPTPRZ is not detected by Western blotting using anti-HNK-1 antibody.^9^ Next, we investigated PTPRZ gene fusion in glioma samples from the C-CAT database (GenMine TOP cancer genome profiling system). Analysis of 301 glioma samples identified 6 *PTPRZ1* gene fusion cases, all of which occur at the *MET* and *PTPRZ1* loci (Figure 5B and Supplementary Figure S5), indicating that the occurrence of PTPRZ-MET gene fusion in glioma is relatively high, 2%. All fusion-positive cases were found in primary glioma samples. The PTPRZ-MET fusion transcripts were found in both IDH mutated and wild-type gliomas, as has been reported.^35^ Multiple types of *PTPRZ-MET* fusion transcripts were observed, with the *PTPRZ1* fusion junction located at exon 1, 2, or 8, while *MET* was consistently fused at exon 2, preserving its kinase domain, as previously described by other groups.^35^^,^^38^ Since the HNK-1 epitope has only been identified at exon 12 of *PTPRZ1*, none of the fusion products detected in the C-CAT database are expected to carry the HNK-1 epitope and are therefore considered PTPRZ-negative.

## Discussion

Differentiating gliomas from PCNSL and other mimicking conditions such as multiple sclerosis (MS) remains a clinical challenge. Despite advancements in imaging technologies, these diseases often present with overlapping radiological features, and definitive diagnosis still requires invasive tissue biopsy. However, since treatment paradigms for gliomas and PCNSL differ fundamentally, there is a clear need for reliable, minimally invasive biomarkers.

In recent years, frequent mutations in MYD88 and CD79B have been identified in PCNSL.^39^ Consequently, liquid biopsy using CSF or blood to check these mutations has garnered increasing attention as a method for differentiating PCNSL,^40^ and they will be promising biomarkers for diagnosis of PCNSL. These molecular assays provide important support for lymphoma diagnosis; however, their sensitivity depends on sufficient tumor-derived DNA and access to specialized molecular testing platforms. It is important to underscore that the primary aim of this study is the development of a biomarker associated with glioma biology rather than lymphoma. In this context, CSF sPTPRZ represents a candidate marker for glioma. Therefore, mutation-based testing for MYD88/CD79B and measurement of CSF sPTPRZ may serve complementary roles in clinical practice: detection of lymphoma-associated mutations may support a diagnosis of PCNSL, whereas elevated CSF sPTPRZ levels may favor a diagnosis of glioma. Such an integrated CSF-based diagnostic approach may enhance clinical decision-making in patients with radiologically ambiguous lesions and improve prebiopsy diagnostic stratification.

Previous studies have demonstrated elevated CSF sPTPRZ levels in glioma patients, distinguishing them from controls and MS.^9^ However, the detection methods employed in those studies relied on IgM antibodies requiring enzymatic digestion, limiting their clinical applicability. In contrast, the anti-HNK-1 IgG antibody used in our study allows robust detection of sPTPRZ without chondroitinase treatment, enabling the development of a clinically practical ELISA system.

Our data demonstrate that CSF sPTPRZ levels were significantly elevated in glioma patients compared with both PCNSL and control groups, supporting its potential as a glioma-associated biomarker. ROC analysis showed strong discrimination between glioma and control (AUC = 0.910, 95% CI: 0.841-0.980) and significant discrimination between glioma and PCNSL (AUC = 0.805, 95% CI: 0.689-0.920). These findings indicate that CSF sPTPRZ provides diagnostic separation not only from nonneoplastic controls but also from PCNSL, a clinically critical differential diagnosis. Notably, stratification by IDH mutation status demonstrated that CSF sPTPRZ levels remained significantly higher than those in PCNSL irrespective of IDH status, suggesting that the elevation is not confined to a single molecular subtype. Subtype-specific analyses demonstrated significant separation between astrocytoma and PCNSL, whereas other glioma subtypes did not reach statistical significance, likely reflecting the limited sample size within individual subgroups. Nevertheless, sPTPRZ elevation was observed across glioma categories. Because CSF from healthy volunteers cannot be ethically obtained, the control cohort comprised patients undergoing clinical evaluation for nontumor neurological conditions. Subgroup analysis did not indicate disproportionate influence of any single diagnosis. However, both the heterogeneity of the control cohort and the limited number of PCNSL cases introduce statistical uncertainty and may affect the precision and stability of performance estimates. Although the observed discrimination and associated confidence intervals suggest potential clinical utility, validation in larger, balanced cohorts will be required to confirm the robustness and generalizability of these findings. These findings further support the potential clinical utility of CSF sPTPRZ as a complementary diagnostic tool in the prebiopsy setting.

Importantly, a subset of glioma patients exhibited low CSF sPTPRZ levels, which initially appeared inconsistent with the overall diagnostic trend. Although we could not directly confirm PTPRZ-MET fusions in these particular cases due to an insufficient number of tumor samples available for RNA analysis, analysis of glioma datasets revealed that such fusions are present in a distinct subset of tumors. Several types of PTPRZ-MET gene fusions exist, generating fusion proteins in which most of the MET kinase domain is preserved.^35^^,^^38^ In a small subset of 4 CSF samples analyzed by Western blotting, 1 case showed undetectable sPTPRZ levels. Consistent with this observation, ELISA analysis of 51 glioma CSF samples demonstrated that approximately 14% of cases had sPTPRZ levels below the diagnostic cut-off. Although this proportion appears higher than the frequency of PTPRZ-MET fusion reported in the C-CAT dataset (6/301), these cohorts are not directly comparable. The Western blot subset was limited in size, and the ELISA cohort was not genomically characterized for fusion status. Furthermore, fusion detection in large-scale sequencing datasets depends on tumor purity, sequencing depth, panel design, and analytical thresholds, which may underestimate certain rearrangements. Recent reports describing alternative PTPRZ fusion partners, including ETV1^36^ and BRAF,^37^ further suggest that the fusion landscape of PTPRZ may be broader than currently appreciated. In addition, fusion-independent mechanisms—such as transcriptional regulation or alterations in ectodomain shedding—may also contribute to reduced sPTPRZ levels. Larger studies integrating genomic and proteomic analyses will be necessary to clarify these possibilities.

Our mass spectrometry analysis of purified sPTPRZ from CSF demonstrated that the HNK-1-capped O-mannose glycan is localized to the juxtamembrane region, which is unique to the PTPRZ-long isoform and consistently absent in all reported PTPRZ-MET fusion variants. This molecular detail offers a plausible explanation for sPTPRZ negativity in some gliomas and reinforces the biomarker’s specificity for intact PTPRZ-long.

To further visualize the region of HNK-1 modification, we generated an AlphaFold model of PTPRZ (Figure 5C).^41^^,^^42^ The model revealed that the modification sites reside in a highly disordered region of the protein, further supporting the hypothesis that the epitope is unique to the full-length form of PTPRZ.

Interestingly, MET inhibitors have shown therapeutic efficacy in tumors with MET fusions in other cancers, such as lung adenocarcinoma.^43^ The presence of PTPRZ-MET fusion in gliomas suggests a similar therapeutic opportunity, as indicated in recent studies.^44^ Thus, detecting fusion-positive gliomas through CSF profiling could serve not only as a diagnostic tool but also to guide targeted therapy.

Additionally, our findings confirm that PTPRZ and its glycosylation machinery—especially GnT-IX—are selectively expressed in gliomas but absent in PCNSL. This supports a glioma-specific glycosylation signature, which may contribute to tumor pathophysiology and therapeutic resistance. Targeting such pathways, as shown with GnT-IX inhibition in previous studies,^16^ may present novel therapeutic opportunities.

Finally, our previous data show that CSF obtained via lumbar puncture is suitable for sPTPRZ quantification,^9^ broadening the test’s application beyond intraoperative settings. This finding emphasizes the feasibility of using CSF sPTPRZ as a minimally invasive biomarker for glioma diagnosis and disease monitoring in outpatient clinical practice.

This study has several limitations, including the modest sample size—particularly for PCNSL—and the heterogeneity of the control cohort. These factors may affect the precision of performance estimates and limit subgroup analyses. In addition, other tumor types that may mimic PCNSL radiologically, such as metastatic carcinoma and melanoma, were not included in the present analysis and should be evaluated in future studies. Larger prospective studies are required to confirm the robustness and generalizability of these findings.

In conclusion, our preliminary findings suggest that sPTPRZ, as detected using an anti-HNK-1 antibody, has the potential to serve as a specific and minimally invasive biomarker for gliomas. Within our cohort, distinguished gliomas from PCNSL and showed potential for identifying PTPRZ-MET fusion-positive cases. Further prospective studies in larger, independent cohorts are warranted to validate these findings and facilitate clinical translation toward more personalized treatment strategies.

## Supplementary Material

vdag101_Supplementary_Data
