# Supplementary material for: Cerebrospinal fluid soluble protein tyrosine phosphatase receptor type Z detected with human natural killer-1 antibody as a practical biomarker for glioma diagnosis
Source: Neurooncol Adv. 2026 Apr 16;8(1):vdag101. doi: 10.1093/noajnl/vdag101 (PMC13175180; doi:10.1093/noajnl/vdag101)
Supplement: vdag101_Supplementary_Data [file vdag101_supplementary_data.docx]

**Supplementary Table 1.**

**Sequences of qPCR primers and probes used in this study**

| **Targeting gene**  **(Protein)** | **Primer and probe sequence (5’-3’)** |
| --- | --- |
| *PTPRZ1* | F: GACTCAGAAATAACTCCTGGATTCC |
| (PTPRZlong) | R: GACCAATACGAGACTCATGGCTA |
|  | FAM-CCTCTGCCTCTGAAACGTGGAACACTTCTG-BHQ-1 |
| *PTPRZ1* | F: TCCTCCAGACAACAGGATTTGG |
| (PTPRZshort) | R: TGGCTACTATTACTGGCCTCATTG |
|  | FAM- ACGGTCAACGTGGTATACTCGCAGACAACC-BHQ-1 |
| *MGAT5B* | F: ACCCTACGAGTACACCTGCG |
| (GnT-IX) | R: GGTAGGGCAGGGTCTGGAG |
|  | FAM-CACGCCTACATCCAGCACCAGGACTTCT-BHQ-1 |
| *18S ribosomal RNA* | F: GCAATTATTCCCCATGAACG |
|  | R: GGGACTTAATCAACGCAAGC |
|  | ProbeLibrary probe 48 (Roche) |

The probes for PTPRZ-long (PTPRZ1 isoforms 1, 4, and 5), PTPRZ-short (PTPRZ1 isoforms 2 and 3) and *MGAT5B* genes were labeled with the fluorescent reporter dye FAM at its 5ʹ end and the quencher dye BHQ-1 at its 3ʹ end. The probe for ribosomal RNA was labeled with VIC at its 5ʹ end and the quencher dye BHQ-1 at its 3ʹ end.

**Supplementary Table 2.**

**Confusion matrix and classification performance at the prespecified threshold (0.31 μg/mL) for glioma versus PCNSL (positive class: glioma)**

**Predicted class**

| Sensitivity (Recall) | 0.863 |
| --- | --- |
| Specificity | 0.600 |
| Precision | 0.880 |
| F1 score | 0.871 |
| Accuracy | 0.803 |

**True class**

|  | sPTPRZ ≧ 0.31 μg/mL | sPTPRZ <  0.31 μg/mL |
| --- | --- | --- |
| Glioma | 44 | 7 |
| PCNSL | 6 | 9 |

**Figure S1. Triple immunofluorescence staining for HNK-1, GFAP, and Olig2 in PCNSL tissue.**

Triple immunofluorescence staining for **HNK-1 (green), GFAP (red), and Olig2 (blue)** in PCNSL tissue sections. Nuclei were counterstained with **DAPI.** Scale bar, 50 μm.


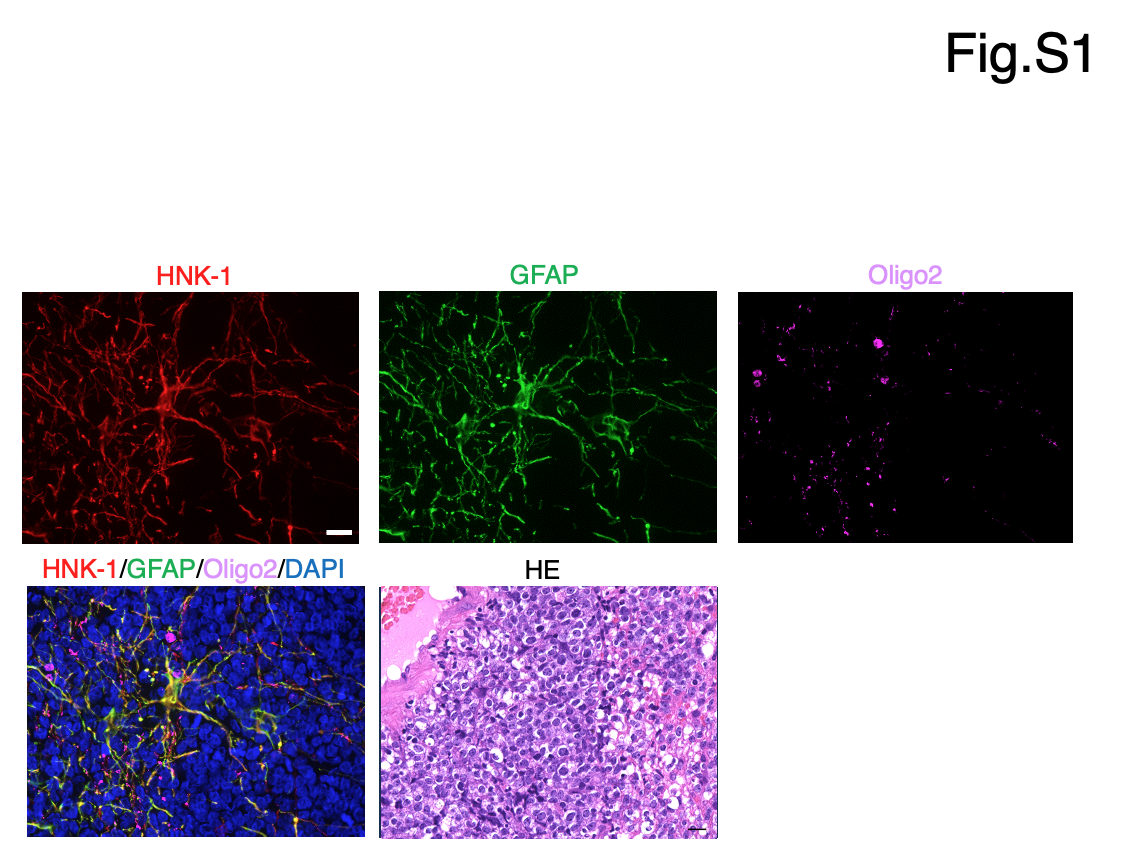


**Figure S2. Comparison of CSF sPTPRZ levels among control subgroups.**

Control subgroup comparisons were performed using the Kruskal–Wallis test among diagnoses with n ≥ 3. Subgroups with n < 3 were displayed descriptively and were not included in statistical testing.


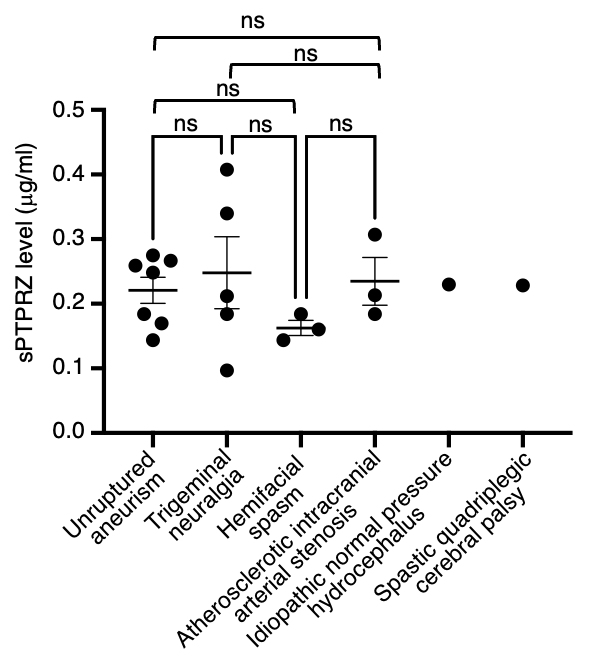


**Figure S3.** Precision–recall curve for discrimination between glioma and PCNSL.

Precision–recall curve for classification of glioma versus PCNSL (positive class: glioma). The area under the precision–recall curve (AUPRC) was 0.94. The dashed line represents the baseline precision corresponding to the prevalence of glioma in the cohort (51/66 = 0.77).


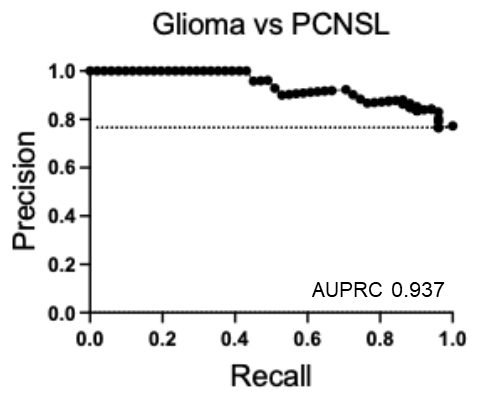


**Figure S4. Annotated tandem mass (MS/MS) spectrum of an HNK-1-carrying glycopeptide.**

The core peptide is DGSVTSTKLLFPSK (residues 1384–1397). Y-ions and B-ions (oxonium ions) generated by glycosidic bond dissociation are annotated and highlighted in blue and orange, respectively. Due to the presence of multiple potential O-glycosylation sites (Ser/Thr) within the peptide and extensive fragmentation of the glycan portion from the peptide backbone, the exact glycosylation site could not be determined. The peptide backbone fragments are shown as b-ions (purple) and y-ions (green).


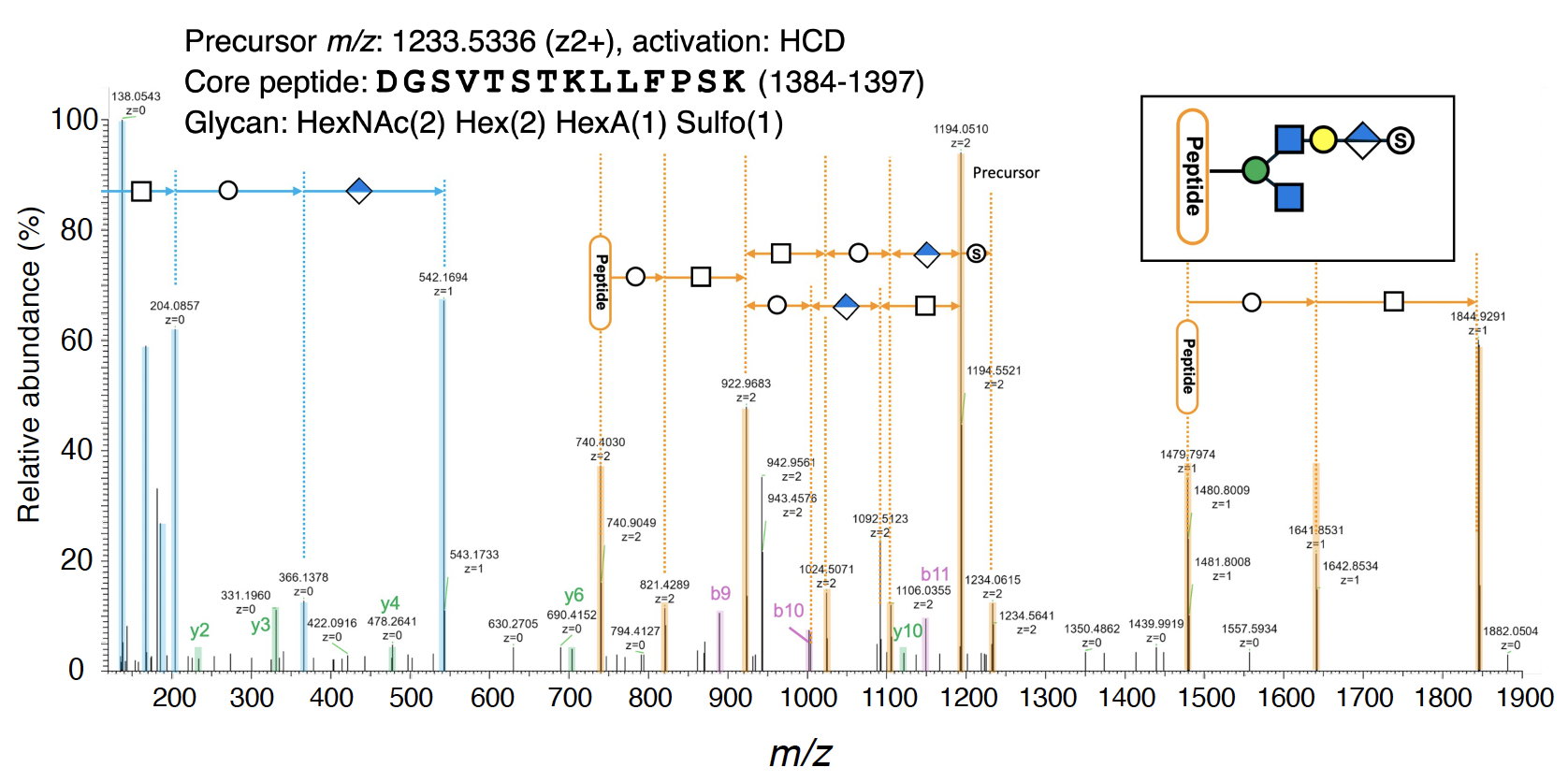


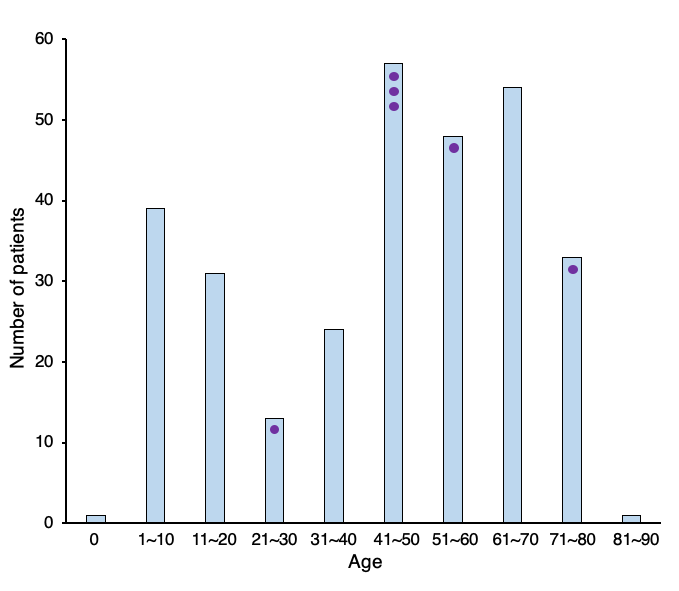
**Figure S5. Age distribution of glioma patients from C-CAT data (GeneMineTOP).**

Blue bars represent the number of patients in each age group. Purple dots indicate individual cases in which the PTPRZ1-MET gene fusion was detected.
